# Heart rate recovery and morbidity after noncardiac surgery: Planned secondary analysis of two prospective, multi-centre, blinded observational studies

**DOI:** 10.1371/journal.pone.0221277

**Published:** 2019-08-21

**Authors:** Gareth L. Ackland, Tom E. F. Abbott, Gary Minto, Martin Clark, Thomas Owen, Pradeep Prabhu, Shaun M. May, Joseph A. Reynolds, Brian H. Cuthbertson, Duminda Wijesundera, Rupert M. Pearse, for the METS and POM-HR Study Investigators

**Affiliations:** 1 William Harvey Research Institute, Queen Mary University of London, London, United Kingdom; 2 Department of Anaesthesia, Derriford Hospital, Plymouth Hospitals NHS Trust; Peninsula Schools of Medicine and Dentistry, Plymouth University, Plymouth, United Kingdom; 3 Department of Anaesthesia, Royal Bournemouth Hospital, Bournemouth, United Kingdom; 4 Department of Anaesthesia, Royal Preston Hospital, Lancashire Teaching Hospitals NHS Foundation Trust, Preston, United Kingdom; 5 Department of Anaesthesia, Royal Surrey County Hospital, Guildford, United Kingdom; 6 Department of Critical Care Medicine, Sunnybrook Health Sciences Centre, Toronto, Ontario, Canada; 7 Department of Anesthesia, University of Toronto, Toronto, Ontario, Canada; 8 Li Ka Shing Knowledge Institute, St. Michael's Hospital, Toronto, Ontario, Canada; 9 Department of Anesthesia and Pain Management, Toronto General Hospital, Toronto, Ontario, Canada; Universidade de Mogi das Cruzes, BRAZIL

## Abstract

**Background:**

Impaired cardiac vagal function, quantified preoperatively as slower heart rate recovery (HRR) after exercise, is independently associated with perioperative myocardial injury. Parasympathetic (vagal) dysfunction may also promote (extra-cardiac) multi-organ dysfunction, although perioperative data are lacking. Assuming that cardiac vagal activity, and therefore heart rate recovery response, is a marker of brainstem parasympathetic dysfunction, we hypothesized that impaired HRR would be associated with a higher incidence of morbidity after noncardiac surgery.

**Methods:**

In two prospective, blinded, observational cohort studies, we established the definition of impaired vagal function in terms of the HRR threshold that is associated with perioperative myocardial injury (HRR ≤ 12 beats min^-1^ (bpm), 60 seconds after cessation of cardiopulmonary exercise testing. The primary outcome of this secondary analysis was all-cause morbidity three and five days after surgery, defined using the Post-Operative Morbidity Survey. Secondary outcomes of this analysis were type of morbidity and time to become morbidity-free. Logistic regression and Cox regression tested for the association between HRR and morbidity. Results are presented as odds/hazard ratios [OR or HR; (95% confidence intervals).

**Results:**

882/1941 (45.4%) patients had HRR≤12bpm. All-cause morbidity within 5 days of surgery was more common in 585/822 (71.2%) patients with HRR≤12bpm, compared to 718/1119 (64.2%) patients with HRR>12bpm (OR:1.38 (1.14–1.67); p = 0.001). HRR≤12bpm was associated with more frequent episodes of pulmonary (OR:1.31 (1.05–1.62);p = 0.02)), infective (OR:1.38 (1.10–1.72); p = 0.006), renal (OR:1.91 (1.30–2.79); p = 0.02)), cardiovascular (OR:1.39 (1.15–1.69); p<0.001)), neurological (OR:1.73 (1.11–2.70); p = 0.02)) and pain morbidity (OR:1.38 (1.14–1.68); p = 0.001) within 5 days of surgery.

**Conclusions:**

Multi-organ dysfunction is more common in surgical patients with cardiac vagal dysfunction, defined as HRR ≤ 12 bpm after preoperative cardiopulmonary exercise testing.

**Clinical trial registry:**

ISRCTN88456378.

## Introduction

Reduced efferent vagal neural activity (hereafter, vagal dysfunction) is a common feature of injury and systemic inflammation.[1, 2] Experimental medicine studies have demonstrated that parasympathetic neurotransmitters released by vagal nerve activity confer organ protection,[3] in part through maintaining exercise capacity[4] and/or limiting systemic inflammation.[5] Preservation, or augmentation, of efferent vagal nerve activity reduces cardiac,[6] pulmonary[7] and renal injury,[8, 9] as well as enhancing endogenous analgesic mechanisms[10] and promoting gastrointestinal recovery after surgery.[11] Slower heart rate recovery (HRR) after exercise is associated with an increased risk of perioperative myocardial injury after noncardiac surgery.[12]

Parasympathetic vagal innervation of the heart can be quantified by heart rate recovery following peak exercise [13–15]; heart rate recovery is independent of exercise workload.[16] Vagal reactivation is the primary mechanism underlying deceleration of heart rate after exercise, as demonstrated by its blockade in humans by atropine.[17] Athletes exhibit accelerated, vagally mediated heart rate recovery after exercise, in contrast to the blunted response observed in patients with heart failure.[17] Heart rate control is attributable to neuronal substrate within the nucleus ambiguus. [18] The other main parasympathetic brainstem component, the dorsal vagal motor nucleus, also regulates cardiac ventricular function, as well as innervating multiple other organs.[18] Developmental ontologic studies show that neurons from the DVMN complex give rise to neurons comprising the nucleus ambiguous.[18] Therefore, dysfunction in nucleus ambiguous neurons, is likely to be mirrored in other vagal neurons.

These data suggest a mechanistic role for cardiac vagal dysfunction in promoting perioperative myocardial injury. Whether cardiac vagal impairment reflects broader parasympathetic impairment that may promote perioperative organ dysfunction has not been systematically explored.

Here, we hypothesized that impaired HRR, a physiologic marker of impaired cardiac parasympathetic activity, was associated with increased postoperative morbidity.

## Methods

### Study design and setting

We undertook a prespecified secondary analysis of two international, multi-centre, prospective observational studies utilising cardiopulmonary exercise testing. In both studies, morbidity data were prospectively collected at the same timepoints after surgery, with both patients and clinicians blinded to heart rate recovery. Using these similar datasets, we assessed whether impaired vagal activity was associated with excess postoperative morbidity after noncardiac surgery. Research ethics committees reviewed both studies, which were conducted in accordance with the principles of the Declaration of Helsinki and the Research Governance Framework.

The Post Operative Morbidity-Heart Rate recovery (POM-HR) study was approved by UK Medical Research Ethical Committee (MREC):12/LO/0453 [approved 30/5/2012]; ISRCTN88456378). The Measurement of Exercise Tolerance before Surgery (METS) study was approved by UK MREC 13/LO/0135 [approved 15 February 2013].

The POM-HR study prospectively recruited high-risk patients (>40y) in 5 UK centres from to 1/11/2012 to 17/1/2015. Inclusion criteria were: (1) referral for cardiopulmonary exercise testing from the patients surgical and/or anaesthesia preassessment clinic; (2) surgery of duration >2 h; (3) at higher risk of complications after surgery, as estimated by their referring anaesthesiologist and/or surgical service. Patients were excluded if they had ATS-defined contraindications to cardiopulmonary exercise testing. The primary outcome in POM-HR was any postoperative complication as defined by the Postoperative Morbidity Survey (POMS)^22^ within five days of surgery.

The METS observational study was not registered; the protocol and methods were published prior to completion of the study. [19, 20] METS was undertaken at 25 hospitals in Canada, UK, Australia, and New Zealand, recruiting patients from March 1, 2013 to March 25, 2016. Inclusion criteria were: (1) participants >40y (2) elective noncardiac surgery under general or regional anaesthesia (or both) with a minimum of one overnight hospital stay (3) ≥1 risk factor for cardiac complications (coronary artery disease, cardiac failure, cerebrovascular disease, diabetes mellitus, chronic renal failure, peripheral arterial disease, hypertension, a history of tobacco smoking within 12 months of surgery, or >70y). Exclusion criteria were: endovascular surgery; insufficient time for cardiopulmonary exercise testing (CPET) before surgery; implantable cardioverter–defibrillator; pregnancy; previous enrolment in the study; severe hypertension (>180/100 mm Hg) and/or other American Thoracic Society-defined contraindications to undertaking CPET. The primary outcome in METS was death or myocardial infarction within 30 days after surgery. The secondary outcomes included complications after surgery, as defined by POMS and Clavien-Dindo grading.

### Cardiopulmonary exercise testing (CPET)

Participants undertook CPET on an electronic cycle ergometer to maximal tolerance, having continued their normal cardiovascular medications up to and including the day of the test. [21] Continuous 12-lead electrocardiogram was recorded. Resting heart rate was recorded before unloaded pedaling in the sitting position. Equipment was calibrated before each test using standard reference gases. Continuous breath-by-breath gas exchange analysis was performed. All patients were instructed to continue cycling until symptom-limited fatigue occurred. After peak effort was reached, workload was reduced to 20W and the participant continued to pedal for five minutes in order to warm-down. Investigators at each site interpreted each CPET and collected a standardised data set (Supplementary data). Clinicians at each site were blinded to the results of cardiopulmonary exercise testing, except where there was a safety concern according to pre-defined criteria.[19] We calculated heart rate recovery by subtracting heart rate 1 minute after the end of exercise from heart rate at peak exercise.[22] Personnel and patients were masked to heart rate recovery data; none of the software used in each centre automatically provided these data. Heart rate recovery data were not provided in reports to clinical teams; therefore these data had no influence on subsequent clinical care.

### Perioperative management

Patients were cared for by the normal attending clinicians, who were blinded to HRR results. All hospitals that contributed patients partake in enhanced recovery programs for the types of surgery involved in this observational study. Surgery and anesthesia were conducted by specialist staff. Perioperative care conformed with local clinical guidelines and was not standardised.

### Exposure of interest

The exposure of interest was heart rate recovery, for which we classified HRR as normal or impaired based on values calculated at 1 minute after the end of peak exercise ≤12 beats.min^-1^. Previous exercise studies that have enrolled >20000 patients in the general population show that HRR ≤12 beats.min^-1^ one minute after cessation of exercise is independently associated with increased mortality. [13, 23]

### Outcomes

The primary outcome was all-cause postoperative morbidity, assessed using the Post Operative Morbidity Survey (POMS; S1 Table), which was collected prospectively within 5 days of surgery.[2] Secondary outcomes were type of morbidity (as defined by POMS), time to become morbidity-free and length of hospital stay.

### Sensitivity analysis

To examine whether cardiac vagal dysfunction is associated with outcomes after surgery independently of subclinical moderate-severe heart failure, [24] we repeated the primary analysis to assess whether the presence of delayed HRR remained associated with the primary outcome in the presence or absence of VO_2_ peak ≤14 ml/kg/min and/or VE/VCO_2_ at the anaerobic threshold ≥34. Preoperative use of beta-blockade, which does not impact negatively on HRR in the cardiac failure population, [24] [25] was also subjected to a similar sensitivity analysis.

### Statistical analysis

Manual and automated validation checks of data were performed both centrally and through source data verification. Descriptive categorical data are summarized as counts (percentage). Descriptive continuous data are presented as mean (95% confidence intervals) and analysed using ANCOVA (controlling for age), with post-hoc Tukey Kramer tests to identify within and between factor differences. We present participants’ characteristics for the whole cohort and stratified by HRR ≤ or >12 beats.min^-1^.

The primary (categorical) outcome was analysed using Fishers exact test for trend. Secondary outcomes were analysed using Fisher’s exact test (type/severity of morbidity) and Cox regression analysis (time-to-become morbidity free), taking into account the following independent variables: age, body-mass index, gender, surgery type (intra-abdominal, orthopaedic, urology/gynaecology, vascular, others), diabetes mellitus, preoperative cardiovascular disease (ischaemia/heart failure/dysrhythmias), resting heart rate and HRR≤12bpm. For log-rank analysis of length of stay, patients who died were right-censored as the largest length of stay. P<0.05 was considered significant. All statistical analyses were undertaken using NCSS 11 (Kaysville, UT, USA).

### Sample size calculation

Sample size was calculated to detect differences in all-cause morbidity on postoperative day 5, assuming that, overall, up to 40% of patients undergoing major surgical procedures may sustain morbidity at this timepoint.[26, 27] On the basis that a 15% relative risk reduction in all-cause morbidity by postoperative day 5 would be of clinical significance, with power of 90%, at least 1920 patients would be required to detect a 15% relative risk reduction in postoperative morbidity comparing patients with HRR≤12 beats min^-1^ versus patients with preserved HRR (α = 0.05).

## Results

### Patient characteristics

**Fig 1:**
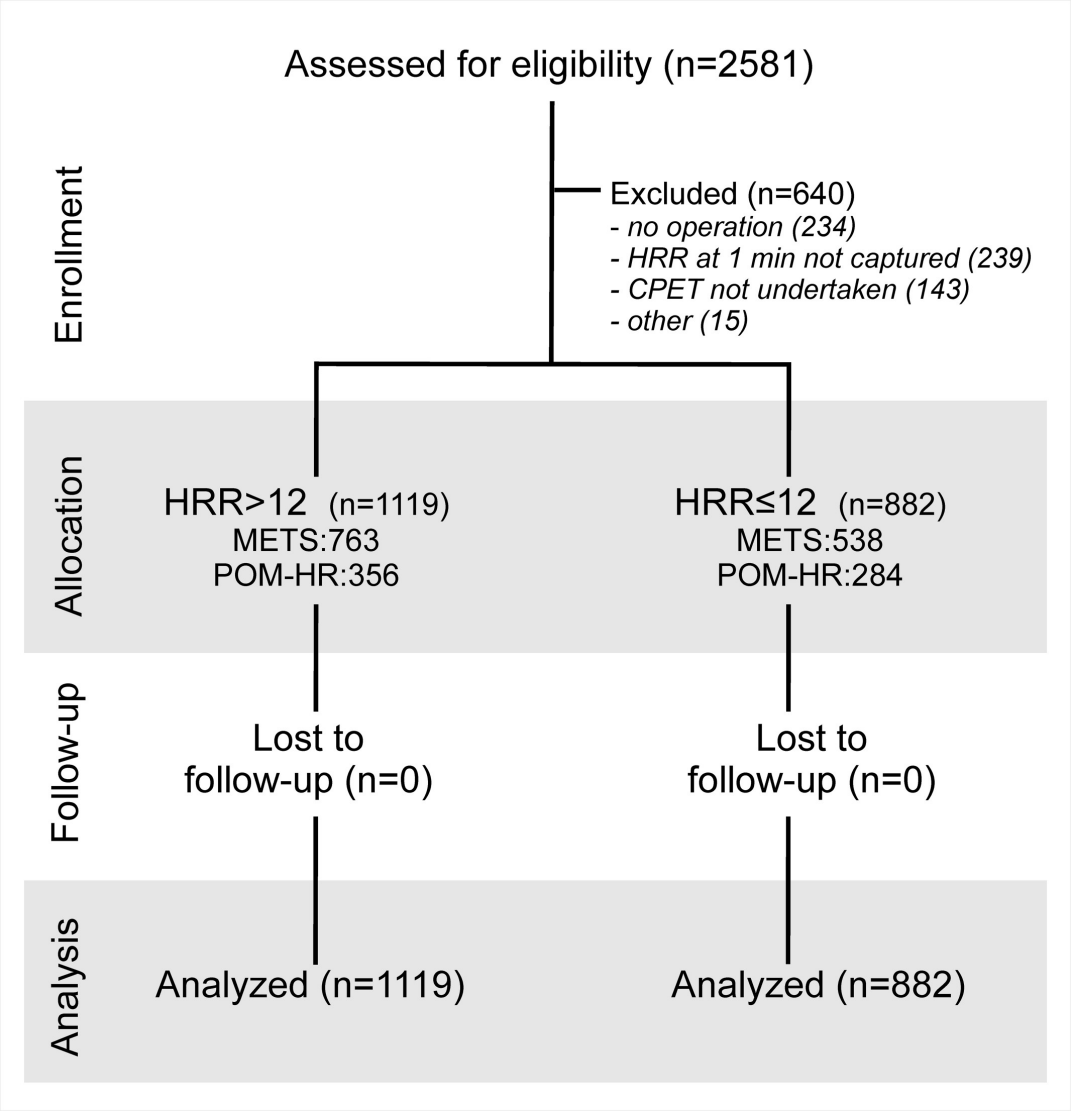
Analysis plan.

**Fig 2:**
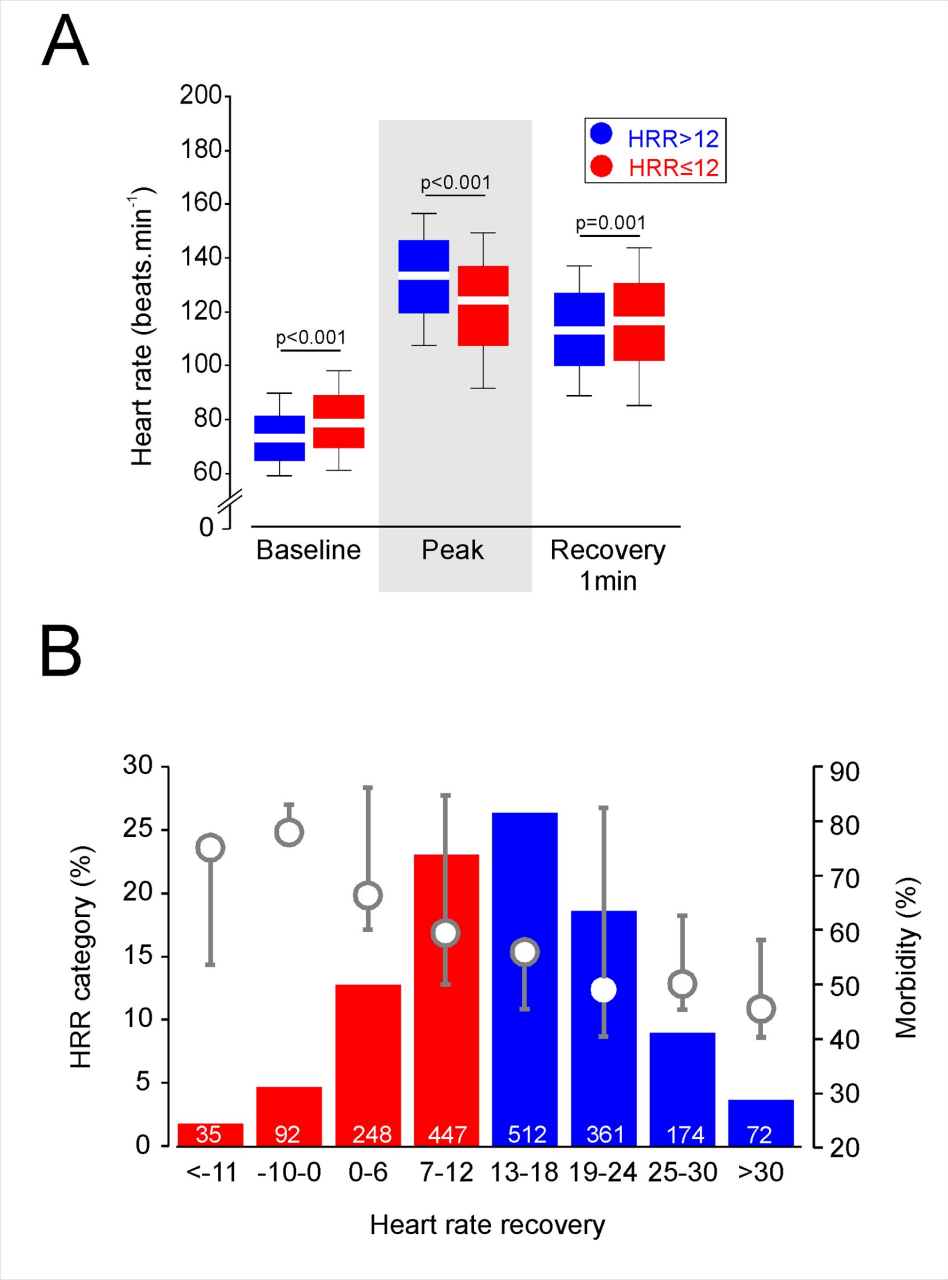
Exercise-evoked heart rate dynamics and distribution of heart rate recovery. **A.** Exercise-evoked changes in heart rate from baseline to peak, and 60s after recovery from the end of exercise in patients with delayed, or normal, heart rate recovery >12beats.min^-1^. Red colour indicate values below which are associated with increased risk of all-cause mortality obtained in large epidemiological studies.[13, 14] Median (25-75^th^ centile values shown; p values determined by ANCOVA, controlling for age. B. Distribution of heart rate recovery (left axis) plotted against all-cause morbidity (right axis) within 5 days of surgery. Red bars indicate values below which are associated with increased risk of all-cause mortality obtained in large epidemiological studies.[13, 14].

1741 patients were recruited into the METS study between 1^st^ March 2013 and 25^th^ March 2016. 840 patients were recruited into the POM-HR study between 1^st^ May 2012 and 31^st^ March 2015. 1941 cases were analysed after cases with missing data were excluded; 882/1941 (45.4%) had HRR≤12 beats min^-1^ (Fig 1). Mean resting heart rate was 6 (95%CI:4–7) beats minute^-1^ higher in patients with HRR≤12 beats min^-1^ (p<0.001, by ANCOVA controlling for age; Fig 2A). Peak heart rate during exercise was 12 (95%CI:10–14) beats minute^-1^ lower in patients with HRR≤12 beats min^-1^ (p<0.001, by ANCOVA controlling for age; Fig 2A). Systolic and diastolic blood pressure at rest were similar (Table 1). Patients with/without delayed heart recovery had similar preoperative characteristics and underwent similar types of surgery (Table 1).

**Table 1:** Patient characteristics.

	HRR>12 (n = 1119)	HRR≤12 (n = 822)
METS (n, %)	763 (68.1%)	538 (65.5%)
Age (years)	64 (11)	68 (10)
Male (n, %)	743 (66%)	487 (59%)
Body mass index (kg.m^-2^)	28.2 (6.0)	28.7 (6.0)
Creatinine (μmol.L^-1^)	77 (67–90)	78 (67–95)
Anaerobic threshold (ml kg min^-1^)	13.0 (4.1)	11.5 (3.3)
Haemoglobin (g.L^-1^)	138 (16)	133 (17)
Systolic blood pressure (mmHg)	128 (17)	130 (19)
Diastolic blood pressure (mmHg)	77 (11)	77 (12)
Beta blocker (n, %)	160 (14%)	197 (24%)
Ca^2+^ channel blocker (n, %)	76 (7%)	93 (11%)
Diuretic (n, %)	50 (4%)	76 (9%)
Anti-platelet (n, %)	216 (19.3%)	141 (16.0%)
ACE-I/ARB (n, %)	322 (29%)	332 (40%)
*Surgical procedure*		
Intra-abdominal (n, %)	478 (43%)	339 (38%)
Orthopaedic (n, %)	180 (16%)	131 (15%)
Urology/gynaecology (n, %)	308 (28%)	227 (26%)
Vascular (n, %)	48 (4%)	45 (5%)
Other (n, %)	104 (9%)	79 (9%)

Data presented as mean (SD), median (25-75^th^ centile), or n (%). % patients/group provided within each surgical category. HRR: heart rate recovery. ACE-I: Angiotensin-converting enzyme inhibitor. ARB: angiotensin receptor blocker.

### Primary outcome: Morbidity within 5 days of surgery

All-cause morbidity within five days of surgery was more common in 585/822 (71.2%) patients with HRR≤12bpm, compared to 718/1119 (64.2%) patients with preserved HRR (OR:1.29 (1.06–1.58); p = 0.001; Table 2; Fig 2B). Morbidity on postoperative day 3 and 5 is provided in S2 Table. Logistic regression analysis showed that lower HRR was progressively independently associated with higher morbidity rates within 5 days of surgery, independent of resting heart rate (S3 and S4 Tables).

**Table 2:** Postoperative morbidity within 5 days of surgery. Data presented as n (%). % patients/HRR group provided within each surgical category. HRR- heart rate recovery.

	HRR>12 (n = 1119)	HRR≤12 (n = 822)	Relative risk (95%CI)	P value
Any POMS morbidity	718 (64.2%)	585 (71.2%)	1.38 (1.14–1.67)	0.001
Pulmonary	220 (19.7%)	199 (24.2%)	1.31 (1.05–1.62)	0.02
Infection	195 (17.4%)	185 (22.5%)	1.38 (1.10–1.72)	0.006
Renal	49 (4.4%)	66 (8.0%)	1.91(1.30–2.79)	<0.001
Gastrointestinal	217 (19.4%)	184 (22.4%)	1.20 (0.96–1.50)	0.11
Cardiovascular	714 (63.8%)	584 (71.0%)	1.39 (1.15–1.69)	<0.001
Neurological	37 (3.3%)	46 (5.6%)	1.73 (1.11–2.70)	0.02
Wound	11 (1.0%)	10 (1.2%)	1.24 (0.52–2.94)	0.66
Blood	17 (1.5%)	21 (2.6%)	1.70 (0.89–3.24)	0.13
Pain	716 (64.0%)	584 (71.0%)	1.38 (1.14–1.68)	0.001

### Secondary outcomes

#### Types of morbidity

HRR≤12 beats.min^-1^ was associated with more frequent episodes of morbidity on both postoperative days 3 and 5. (S2 Table) Cardiovascular morbidity was more common in patients with HRR≤12 beats.min^-1^ (OR:1.39 (1.15–1.69); p<0.001)), characterised by more episodes of hypotension (OR:1.71 (1.06–2.76); p = 0.03), more frequent intraoperative use of norepinephrine (OR:3.71 (1.54–8.94); p = 0.003) and a higher proportion of arrythmias detected independent of hypotension (4.1% versus 3.2% respectively). Patients with HRR≤12 beats.min^-1^ sustained more pulmonary (OR:1.31 (1.05–1.62); p = 0.02), including pneumonia (OR:2.52 (1.05–6.05)), infection (OR:1.38 (1.10–1.72); p = 0.006), renal (OR:1.91 (1.30–2.79); p = 0.02), neurological (OR:1.73 (1.11–2.70); p = 0.02) and pain morbidity (OR:1.38 (1.14–1.68); p = 0.001; S2 Table).

#### Time to become morbidity-free

**Fig 3:**
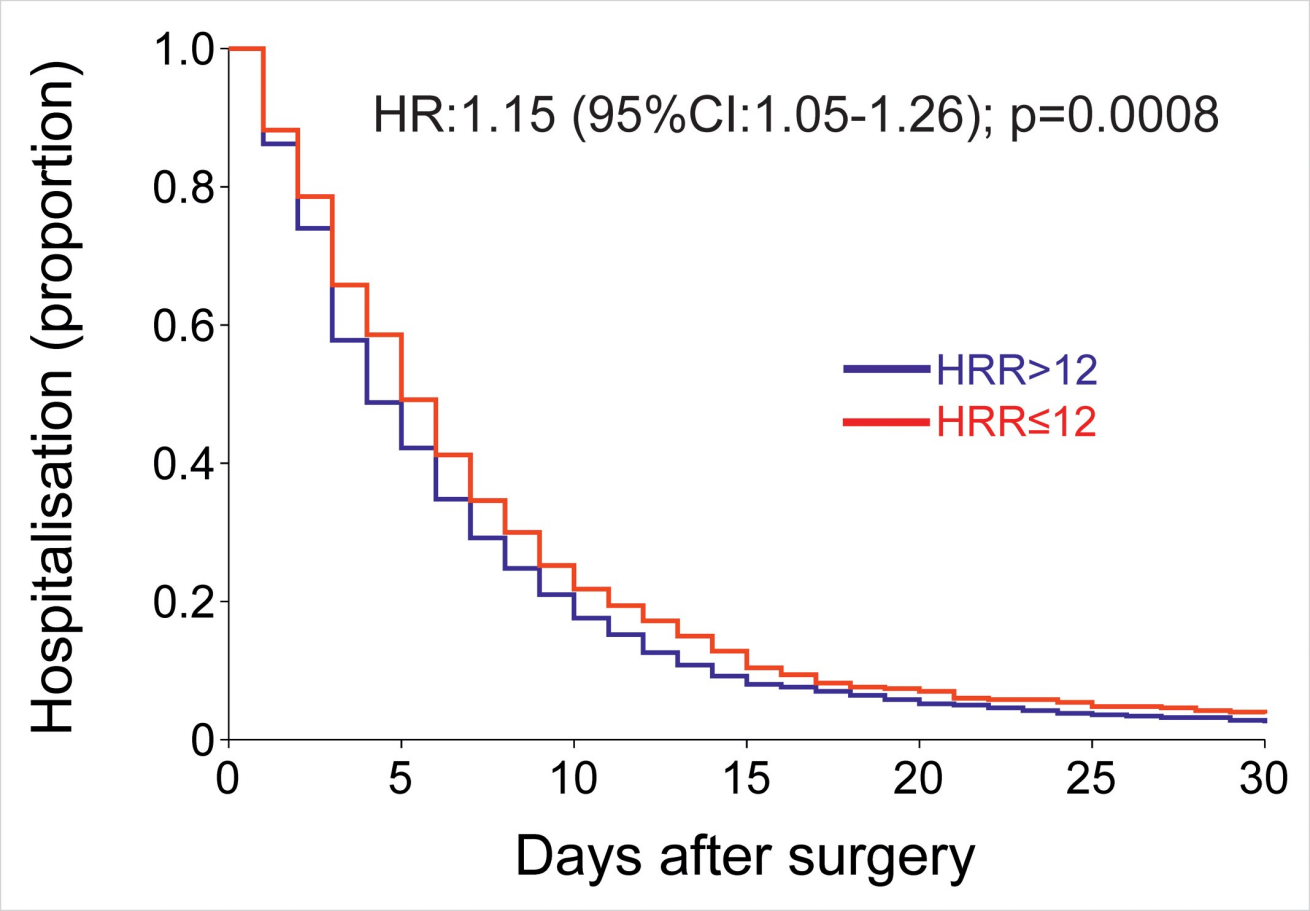
Delayed heart rate and postoperative outcome. Unadjusted Kaplan-Meier estimator for all surgery types showed that delayed heart rate recovery was associated with prolonged hospital stay (HR: 1.15 (95%CI:1.05–1.26); p = 0.0008).

Fewer patients with HRR≤12 beats.min^-1^ (237/882 (28.8%)) remained morbidity-free within 5 days of surgery, compared to 401/1119 (35.8%) patients with preserved HRR (OR:1.35 (1.06–1.70); p = 0.01; S5 Table). Surgery for malignancy (risk ratio:1.26 (1.13–1.40); p<0.001) and HRR≤12 beats.min^-1^ (risk ratio:1.11 (1.05–1.18); p<0.001) were independently associated with delayed hospital discharge (Fig 3; S5 Table). HRR was dose-dependently associated with length of hospital stay (S6 Table).

### Sensitivity analysis

Patients with HRR≤12 beats.min^-1^ remained more likely to sustain morbidity within 5 days of surgery (OR:1.38 (1.12–1.69); p<0.001; S1 Fig), independent of the presence/absence of negatively prognostic CPET factors VO_2_ peak ≤14 ml kg min and/or VE/VCO_2_ ratio ≥34.[28] A similar relationship was observed for patients receiving beta-blockers (OR:1.24 (0.83–1.86). Mortality was 0.7% in patients with preoperative HRR <12, compared to 0.3% in those with HRR>12 (odds ratio:2.7 (95%CI:0.7–10.7)).

## Discussion

The principal finding of these two large generalisable studies is that parasympathetic dysfunction–as quantified by delayed HRR after preoperative exercise- is more frequently associated with morbidity within 5 days of surgery. The broad range, and temporal pattern, of morbidity after surgery is consistent with laboratory/translational work highlighting that loss of vagal activity promotes multiple organ dysfunction. These data reinforce prognostic studies from the general population, where delayed HRR ≤12 beats.minute^-1^ is a strong independent predictor of all-cause mortality in apparently otherwise healthy middle-aged subjects.[13, 14] Taken together, these data suggest that cardiac vagal dysfunction represents a distinct endotype for morbidity after major surgery.

Vagal reactivation is the primary mechanism underlying deceleration of heart rate after exercise, as demonstrated by its blockade in humans by atropine.[17] Athletes exhibit accelerated, vagally mediated heart rate recovery after exercise, in contrast to the blunted response observed in patients with heart failure.[17] Heart rate control is attributable to neuronal substrate within the nucleus ambiguous. The other main parasympathetic brainstem substrate, the dorsal vagal motor nucleus, also regulates cardiac ventricular function, as well as innervating multiple other organs.[4] The development of morbidity across multiple organs suggests that a broad deficiency in parasympathetic neurons may be present in many patients. However, we cannot rule out that many of the morbidities over-represented in patients with delayed heart rate recovery are linked indirectly through non-neural mechanisms. For example, hypotension is more common in patients with delayed heart rate recovery, which in turn may predispose to acute renal dysfunction.[29] Nevertheless, parasympathetic neural activity contributes to a diverse range of physiological functions, with a broader role being plausible in pathological states. Laboratory data shows that vagal neuromodulation of cardiomyocyte, gastrointestinal, renal, lung, neurological and immune cells limits tissue injury in a range of pathological settings. [7, 30, 31] Lack of cardiac vagal activity promotes arrhythmias,[32] perhaps in part due to the detrimental effects of ongoing systemic inflammation.[33] Further reductions in parasympathetic activity induced by anaesthetic agents,[34] opioid analgesics[35] and inflammatory modulation of afferent autonomic inputs[36] following surgery may therefore contribute to postoperative morbidity.[2] Our data support the findings of a small study in patients undergoing thoracic surgery for lung cancer, where a threshold value of HRR ≤12-beat.min-1 following the six-minute walk test was associated with postoperative cardiopulmonary complications.[37]

In cardiac failure, loss of parasympathetic activity is a consistent predictor of accelerated mortality.[38] Failure to improve autonomic function- despite optimal medical therapy- is associated with higher mortality.[39] Patients presenting for major surgery, who are frequently deconditioned as a result of cancer and/or systemic inflammation,[40] appear to share similar strikingly similar pathophysiological features.[28] Impaired heart rate recovery is independent of clinically-defined cardiac comorbidity and is not influenced by drug therapy including continuation of β-blockade.[12, 40]

A strength of this study is the blinding of HRR data to participants and clinicians. The prospective, international multi-centre study design suggests that these data are generalisable. Our findings are limited by the morbidity measure used, given the sensitivity of the POMS for many morbidities after major surgery. This limitation was partly addressed by tracking serial morbidity, which allowed us to assess time to become morbidity free. This measure is more mechanistically informative, as it reflects morbidity over time- as opposed to a snapshot primary outcome which may be misleading. The systematic grading of severity was captured partially for some POMS-domains, and is likely to have provided a richer, more granular understanding. The use of organ-specific biomarkers, as we have found with high-sensitivity troponin,[12] may reveal further mechanistic insights. The choice of HRR threshold, and timepoint after cessation of exercise, requires further study. However, we used a standardised exercise protocol, combined with the most stringent and widely adopted threshold of HRR described thus far.[41] Moreover, in mechanistic work, we found a strong association between the HRR threshold used and a pathological cellular phenotype.[42]

In summary, delayed HRR is independently associated with excess postoperative morbidity leading to prolonged admission. Preoperative parasympathetic dysfunction represents a distinct endotype for morbidity after major surgery.

## Supporting information

S1 File Minimum dataset: Primary outcomes. (XLSX)

S1 Table POMS-defined morbidity. (DOCX)

S2 Table POMS-defined morbidity on postoperative days 3 and 5. (DOCX)

S3 Table Factors associated with patients being free of morbidity within 5 days of surgery. (DOCX)

S4 Table Relationship between magnitude of HRR and factors associated with patients being free of morbidity within 5 days of surgery. (DOCX)

S5 Table Factors associated with delayed discharge from hospital after surgery. (DOCX)

S6 Table Relationship between magnitude of HRR and other factors associated with more rapid discharge from hospital after surgery. (DOCX)

S1 Fig Serial changes in POMS-defined morbidity on postoperative days 3 and 5. (DOCX)

S2 Fig Delayed heart rate recovery and CPET markers of severe cardiac failure. (DOCX)

S3 Fig Delayed heart rate and postoperative outcome. (DOCX)
