# Correction: Heart rate recovery and morbidity after noncardiac surgery: Planned secondary analysis of two prospective, multi-centre, blinded observational studies

**DOI:** 10.1371/journal.pone.0226379

**Published:** 2019-12-05

**Authors:** Gareth L. Ackland, Tom E. F. Abbott, Gary Minto, Martin Clark, Thomas Owen, Pradeep Prabhu, Shaun M. May, Joseph A. Reynolds, Brian H. Cuthbertson, Duminda Wijeysundera, Rupert M. Pearse, for the METS and POM-HR Study Investigators

The tenth author's name is spelled incorrectly. The correct name is: Duminda Wijeysundera.[1]
