# Supplementary material for: Heart rate recovery and morbidity after noncardiac surgery: Planned secondary analysis of two prospective, multi-centre, blinded observational studies
Source: PLoS One. 2019 Aug 21;14(8):e0221277. doi: 10.1371/journal.pone.0221277 (PMC6703687; doi:10.1371/journal.pone.0221277)
Supplement: S1 Table — (DOCX) [file pone.0221277.s002.docx]

# Supplementary Table 1. POMS-defined morbidity.

| **Morbidity type** | **Criteria** |
| --- | --- |
| Pulmonary | *De novo* requirement for supplemental oxygen or other respiratory support (e.g., CPAP or IPPV) |
|  |  |
|  |  |
| Infectious | Currently on antibiotics or temperature >38°C in the last 24 hrs |
|  |  |
| Renal | Presence of oliguria (<500 mL/day), increased serum creatinine (>30% from baseline value), or urinary catheter in place for a non-surgical reasons |
|  |  |
|  |  |
| Gastrointestinal | Unable to tolerate an enteral diet (either by mouth or feeding tube) for any reason, including nausea, vomiting and abdominal distension |
|  |  |
| Cardiovascular | Diagnostic test or therapy in last 24 hrs for any of the following reasons: *de novo* myocardial infarction or ischemia, hypotension (requiring drug therapy or fluid >200 mL/hr), atrial or ventricular arrhythmia or pulmonary edema |
|  |  |
|  |  |
|  |  |
| Neurological | Presence of a *de novo* focal deficit, coma or confusion/delirium |
|  |  |
| Wound complications | Wound dehiscence requiring surgical exploration or drainage or pus from the wound |
|  |  |
| Haematological | Requirement for any of the following within last 24 hrs: blood, platelets, fresh frozen plasma or cryoprecipitate |
| Pain | Surgical wound pain significant enough to require parenteral opiates or regional anesthesia |
