# Supplementary material for: Heart rate recovery and morbidity after noncardiac surgery: Planned secondary analysis of two prospective, multi-centre, blinded observational studies
Source: PLoS One. 2019 Aug 21;14(8):e0221277. doi: 10.1371/journal.pone.0221277 (PMC6703687; doi:10.1371/journal.pone.0221277)
Supplement: S2 Table — (DOCX) [file pone.0221277.s003.docx]

# Supplementary Table 2. POMS-defined morbidity on postoperative days 3 and 5.

|  | **Postoperative day 3** | |  | **Postoperative day 5** | |  |
| --- | --- | --- | --- | --- | --- | --- |
|  | **HRR>12** | **HRR≤12** | **RR (95%CI)** | **HRR>12** | **HRR≤12** | **RR (95%CI)** |
| Any POMS morbidity | 716 | 585 | 1.39 (1.14-1.69) | 295 | 260 | 1.29 (1.06-1.58) |
| Pulmonary | 204 | 184 | 1.30 (1.04-1.63) | 89 | 78 | 1.21 (0.88-1.67) |
| Infection | 155 | 144 | 1.32 (1.03-1.69) | 136 | 124 | 1.28 (0.99-1.67) |
| Renal | 41 | 56 | 1.92 (1.27-2.91) | 24 | 40 | 2.33 (1.40-3.90) |
| Gastrointestinal | 190 | 154 | 1.13 (0.89-1.42) | 133 | 110 | 1.14 (0.87-1.50) |
| Cardiovascular | 713 | 584 | 1.40 (1.15-1.70) | 30 | 33 | 1.52 (0.92-2.51) |
| Neurological | 28 | 34 | 1.68 (1.01-2.80) | 16 | 23 | 1.98 (1.04-3.78) |
| Wound | 2 | 5 | 3.42 (0.66-17.7) | 10 | 7 | 0.95 (0.36-2.51) |
| Blood | 13 | 16 | 1.69 (0.81-3.53) | 5 | 8 | 2.19 (0.71-6.72) |
| Pain | 716 | 584 | 1.38 (1.14-1.68) | 150 | 123 | 1.14 (0.88-1.47) |
