# Supplementary material for: Heart rate recovery and morbidity after noncardiac surgery: Planned secondary analysis of two prospective, multi-centre, blinded observational studies
Source: PLoS One. 2019 Aug 21;14(8):e0221277. doi: 10.1371/journal.pone.0221277 (PMC6703687; doi:10.1371/journal.pone.0221277)
Supplement: S3 Table — (DOCX) [file pone.0221277.s004.docx]

# Supplementary Table 3. Factors associated with patients being free of morbidity within 5 days of surgery.

| **Independent Variable** | **Regression Coefficient** | **OR** | **95%CIs** |  | **P value** |
| --- | --- | --- | --- | --- | --- |
| Age (years) | 0.005 | 1.005 | 0.997 - | 1.012 | 0.232 |
| Body-mass index (kg.m^2^) | 0.004 | 1.004 | 0.991 - | 1.018 | 0.520 |
| Gender (male) | -0.048 | 0.954 | 0.881 - | 1.033 | 0.242 |
| HRR>12 beats.minute^-1^ | -0.099 | 0.906 | 0.836 - | 0.981 | 0.015 |
| Diabetes mellitus | 0.051 | 1.053 | 0.954 - | 1.161 | 0.307 |
| Coronary artery disease | -0.089 | 0.915 | 0.783 - | 1.068 | 0.258 |
| Current malignancy | 0.015 | 1.015 | 0.940 - | 1.097 | 0.701 |
| Procedure type | -0.042 | 0.959 | 0.931 - | 0.988 | 0.006 |
