# Supplementary material for: Heart rate recovery and morbidity after noncardiac surgery: Planned secondary analysis of two prospective, multi-centre, blinded observational studies
Source: PLoS One. 2019 Aug 21;14(8):e0221277. doi: 10.1371/journal.pone.0221277 (PMC6703687; doi:10.1371/journal.pone.0221277)
Supplement: S4 Table — (DOCX) [file pone.0221277.s005.docx]

# Supplementary Table 4. Relationship between magnitude of HRR and factors associated with patients being free of morbidity within 5 days of surgery.

HRR was progressively independently associated with more POMS-defined morbidity within 5 days of surgery. Reference is absence of condition, unless stated otherwise. The default recoding scheme used for translating a categorical variable into a set of internal numeric variables was produced by comparing each categorical variable with the next variable in the dataset. For HRR, lower odds ratio refers to preceding category being more likely to not sustain morbidity.

| **Independent Variable** | **OR** | **Lower 95%CI** | **Upper 95%CI** | **p value** |
| --- | --- | --- | --- | --- |
| Age | 1.00 | 0.99 | 1.01 | 0.846 |
| Body-mass index | 0.99 | 0.97 | 1.01 | 0.280 |
| Heart rate (beats.minute^-1^) | 1.00 | 0.99 | 1.01 | 0.799 |
| Gender | 1.18 | 1.03 | 1.34 | 0.014 |
| Cardiac history | 0.92 | 0.72 | 1.17 | 0.502 |
| Surgical procedure (first comparator: abdominal) | | |  |  |
| *Orthopaedic* | 2.21273 | 1.672 | 2.92827 | <0.001 |
| *Other* | 2.429 | 1.512 | 3.90158 | <0.001 |
| *Urology/gynaecology* | 0.65231 | 0.352 | 1.21015 | 0.175 |
| *Vascular* | 0.25632 | 0.118 | 0.55548 | 0.001 |
| Active malignancy | 2.36 | 1.84 | 3.03 | <0.001 |
| Diabetes mellitus | 1.21 | 1.03 | 1.43 | 0.018 |
| Heart rate recovery (first comparator: HRR>19) | | |  |  |
| *HRR:13-18* | 0.84 | 0.69 | 1.02 | 0.081 |
| *HRR:7-12* | 0.72 | 0.55 | 0.93 | 0.012 |
| *HRR:<6* | 0.69 | 0.55 | 0.87 | 0.002 |
