# Supplementary material for: Heart rate recovery and morbidity after noncardiac surgery: Planned secondary analysis of two prospective, multi-centre, blinded observational studies
Source: PLoS One. 2019 Aug 21;14(8):e0221277. doi: 10.1371/journal.pone.0221277 (PMC6703687; doi:10.1371/journal.pone.0221277)
Supplement: S5 Table — (DOCX) [file pone.0221277.s006.docx]

# Supplementary Table 5. Factors associated with delayed discharge from hospital after surgery.

| **Independent Variable** | **Regression Coefficient** | **RR** | **95%CIs** |  | **P value** |
| --- | --- | --- | --- | --- | --- |
| Age (years) | -0.002 | 0.998 | 0.992 - | 1.004 | 0.505 |
| Body-mass index (kg.m^2^) | 0.006 | 1.006 | 0.996 - | 1.015 | 0.255 |
| Gender (male) | 0.024 | 1.024 | 0.966 - | 1.085 | 0.423 |
| HRR>12 beats.minute^-1^ | -0.219 | 0.804 | 0.716 - | 0.902 | <0.001 |
| Diabetes mellitus | 0.018 | 1.018 | 0.879 - | 1.178 | 0.814 |
| Coronary artery disease | 0.073 | 1.076 | 0.865 - | 1.338 | 0.512 |
| Procedure type | -0.487 | 0.615 | 0.538 - | 0.702 | <0.001 |
| Current malignancy | 0.168 | 1.183 | 1.120 - | 1.249 | <0.001 |
