# Supplementary material for: Heart rate recovery and morbidity after noncardiac surgery: Planned secondary analysis of two prospective, multi-centre, blinded observational studies
Source: PLoS One. 2019 Aug 21;14(8):e0221277. doi: 10.1371/journal.pone.0221277 (PMC6703687; doi:10.1371/journal.pone.0221277)

# Supplementary Figure 1. Serial changes in POMS-defined morbidity on postoperative days 3 and 5.

**Chi-Square Prob Reject H0**

**Test Type Value DF Level at α = 0.05?**

Likelihood Ratio 2-Sided 24.5577 14 0.03919 Yes

Fisher's Exact


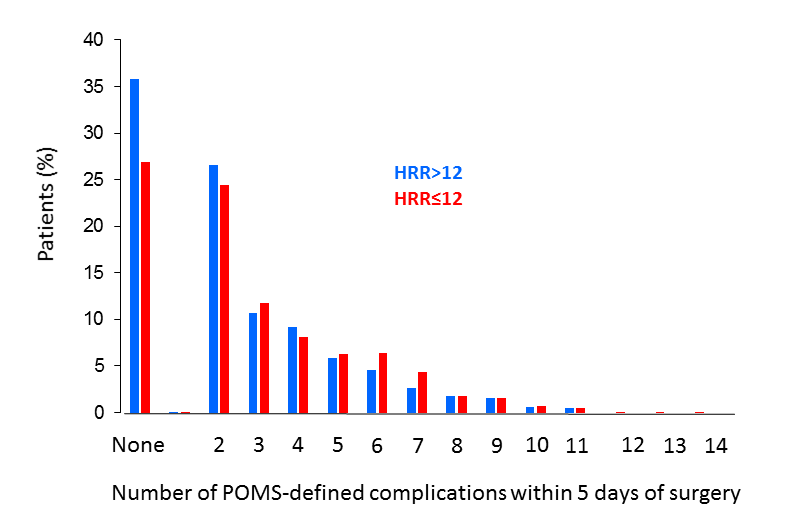


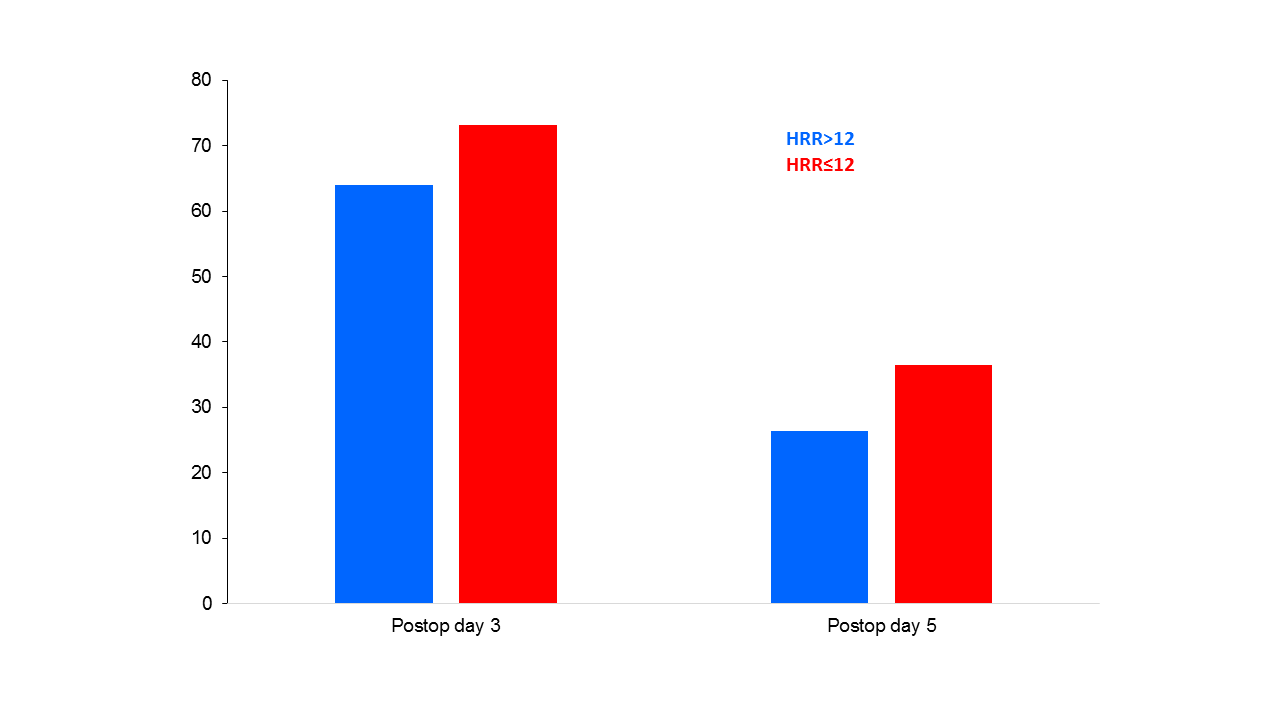

Supplement: S1 Fig — (DOCX) [file pone.0221277.s008.docx]
