# Supplementary material for: Heart rate recovery and morbidity after noncardiac surgery: Planned secondary analysis of two prospective, multi-centre, blinded observational studies
Source: PLoS One. 2019 Aug 21;14(8):e0221277. doi: 10.1371/journal.pone.0221277 (PMC6703687; doi:10.1371/journal.pone.0221277)

# Supplementary Figure 2. Delayed heart rate recovery and CPET markers of severe cardiac failure.

Patients with HRR≤12 beats.min^-1^ remained more likely to sustain morbidity within 5 days of surgery (OR:1.38 (1.12-1.69); p<0.001; Supplementary data), independent of the presence/absence of negatively prognostic CPET factors VO_2_ peak ≤14ml kg min and/or VE/VCO_2_ ratio ≥34.

#
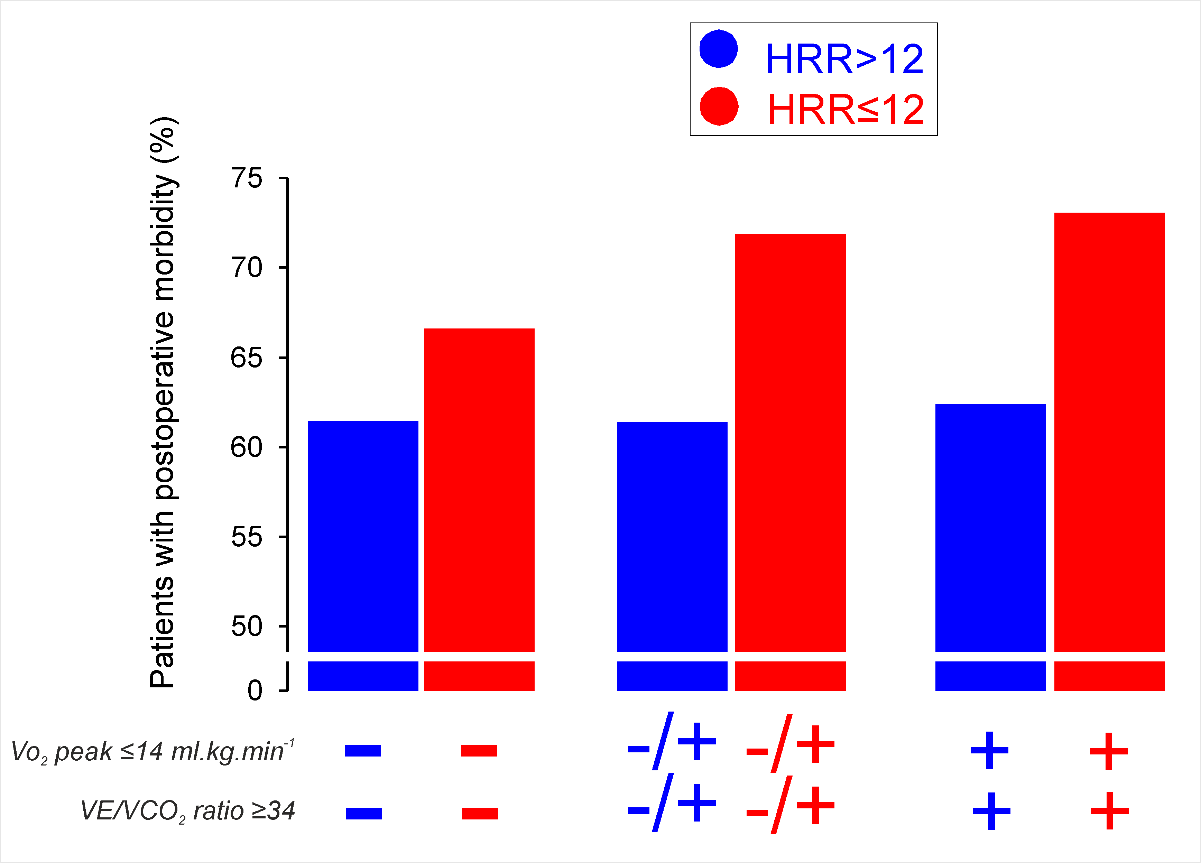

Supplement: S2 Fig — (DOCX) [file pone.0221277.s009.docx]
