# Supplementary material for: Heart rate recovery and morbidity after noncardiac surgery: Planned secondary analysis of two prospective, multi-centre, blinded observational studies
Source: PLoS One. 2019 Aug 21;14(8):e0221277. doi: 10.1371/journal.pone.0221277 (PMC6703687; doi:10.1371/journal.pone.0221277)
Supplement: S3 Fig — (DOCX) [file pone.0221277.s010.docx]

# Supplementary Figure 3. Delayed heart rate and postoperative outcome.


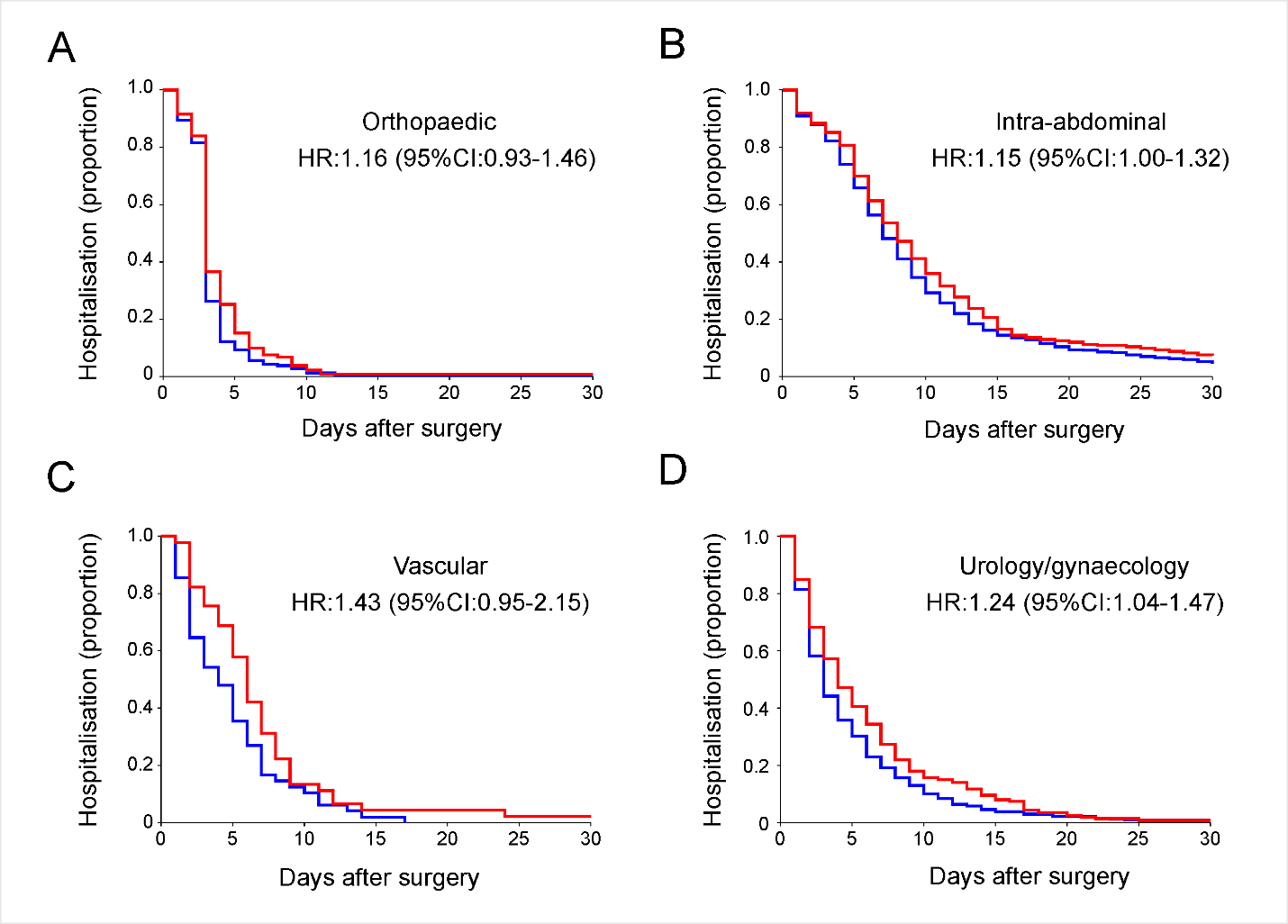
Unadjusted log-rank analysis for all surgery types showed that delayed heart rate recovery was associated with prolonged hospital stay (HR: 1.15 (95%CI:1.05-1.26); p=0.0008). Length of hospital stay are shown for individual surgical categories: A. orthopaedic (n=311); B. intra-abdominal (n=817); C. vascular (n=93); D. urology/gynaecology (n=535).
